# Laparoscopic peritoneal lavage versus sigmoidectomy for perforated diverticulitis with purulent peritonitis: three-year follow-up of the randomised LOLA trial

**DOI:** 10.1007/s00464-022-09326-3

**Published:** 2022-05-23

**Authors:** Vincent T. Hoek, Pim P. Edomskis, Pieter W. Stark, Daniel P. V. Lambrichts, Werner A. Draaisma, Esther C. J. Consten, Johan F. Lange, Willem A. Bemelman, The LADIES trial collaborators, W C Hop, B C Opmeer, J B Reitsma, R A Scholte, E W H Waltmann, A Legemate, J F Bartelsman, D W Meijer, M de Brouwer, J van Dalen, M Durbridge, M Geerdink, G J Ilbrink, S Mehmedovic, P Middelhoek, M J Boom, E C J Consten, J D W van der Bilt, G D J van Olden, M A W Stam, M S Verweij, Sandra Vennix, Gijsbert D Musters, Hilko A Swank, Marja A Boermeester, O R C Busch, C J Buskens, Y El-Massoudi, A B Kluit, C C van Rossem, M P Schijven, P J Tanis, C Unlu, Susan van Dieren, M F Gerhards, T M Karsten, L C de Nes, H Rijna, B A van Wagensveld, G I Koff eman, E P Steller, J B Tuynman, S C Bruin, D L van der Peet, C F J M Blanken-Peeters, H A Cense, E Jutte, R M P H Crolla, G P van der Schelling, M van Zeeland, E J R de Graaf, R P R Groenendijk, T M Karsten, M Vermaas, O Schouten, M R de Vries, H A Prins, D J Lips, R J I Bosker, J A B van der Hoeven, J Diks, P W Plaisier, P M Kruyt, C Sietses, M W J Stommel, S W Nienhuijs, I H J T de Hingh, M D P Luyer, G van Montfort, E H Ponten, J F Smulders, E B van Duyn, J M Klaase, D J Swank, R T Ottow, H B A C Stockmann, J Vermeulen, R J C L M Vuylsteke, H J Belgers, S Fransen, E M von Meijenfeldt, M N Sosef, A A W van Geloven, E R Hendriks, B ter Horst, M M N Leeuwenburgh, O van Ruler, J M Vogten, E J C Vriens, M Westerterp, Q A J Eijsbouts, A Bentohami, T S Bijlsma, N de Korte, D Nio, M J P M Govaert, J J A Joosten, R A E M Tollenaar, L P S Stassen, M J Wiezer, E J Hazebroek, A B Smits, H L van Westreenen, J F Lange, A Brandt, W N Nijboer, Irene M Mulder, B R Toorenvliet, W F Weidema, P P L O Coene, G H H Mannaerts, D den Hartog, R J de Vos, J F Zengerink, A G M Hoofwijk, K W E Hulsewé, J Melenhorst, J H M B Stoot, W H Steup, P J Huijstee, J W S Merkus, J J Wever, J K Maring, J Heisterkamp, W M U van Grevenstein, M R Vriens, M G H Besselink, I H M Borel Rinkes, A J Witkamp, G D Slooter, J L M Konsten, A F Engel, E G J M Pierik, T G Frakking, D van Geldere, G A Patijn, Belgium A J L D’Hoore, A van Overstraeten de Buck, M Miserez, I Terrasson, A Wolthuis, S di Saverio, M G de Blasiis

**Affiliations:** 1 Department of Surgery, Erasmus University Medical Centre, Rotterdam, The Netherlands; 2 Department of Surgery, Jeroen Bosch Hospital, Den Bosch, The Netherlands; 3 Department of Surgery, Meander Medical Centre, Amersfoort, The Netherlands; 4 Department of Surgery, University Medical Centre Groningen, Groningen, The Netherlands; 5 Department of Surgery, IJsselland Hospital, Capelle aan den IJssel, The Netherlands; 6 Department of Surgery, Amsterdam University Medical Centre, University of Amsterdam, Amsterdam, The Netherlands; 7 Department of Surgery, Erasmus University Medical Center, Wytemaweg 80 3015 CN Room Ee-173, Rotterdam, The Netherlands

**Keywords:** Laparoscopic lavage, Complicated diverticulitis

## Abstract

**Background:**

This study aimed to compare laparoscopic lavage and sigmoidectomy as treatment for perforated diverticulitis with purulent peritonitis during a 36 month follow-up of the LOLA trial.

**Methods:**

Within the LOLA arm of the international, multicentre LADIES trial, patients with perforated diverticulitis with purulent peritonitis were randomised between laparoscopic lavage and sigmoidectomy. Outcomes were collected up to 36 months. The primary outcome of the present study was cumulative morbidity and mortality. Secondary outcomes included reoperations (including stoma reversals), stoma rates, and sigmoidectomy rates in the lavage group.

**Results:**

Long-term follow-up was recorded in 77 of the 88 originally included patients, 39 were randomised to sigmoidectomy (51%) and 38 to laparoscopic lavage (49%). After 36 months, overall cumulative morbidity (sigmoidectomy 28/39 (72%) versus lavage 32/38 (84%), *p* = 0·272) and mortality (sigmoidectomy 7/39 (18%) versus lavage 6/38 (16%), *p* = 1·000) did not differ. The number of patients who underwent a reoperation was significantly lower for lavage compared to sigmoidectomy (sigmoidectomy 27/39 (69%) versus lavage 17/38 (45%), *p* = 0·039). After 36 months, patients alive with stoma in situ was lower in the lavage group (proportion calculated from the Kaplan–Meier life table, sigmoidectomy 17% vs lavage 11%, log-rank *p* = 0·0268). Eventually, 17 of 38 (45%) patients allocated to lavage underwent sigmoidectomy.

**Conclusion:**

Long-term outcomes showed that laparoscopic lavage was associated with less patients who underwent reoperations and lower stoma rates in patients alive after 36 months compared to sigmoidectomy. No differences were found in terms of cumulative morbidity or mortality. Patient selection should be improved to reduce risk for short-term complications after which lavage could still be a valuable treatment option.

**Graphical abstract:**

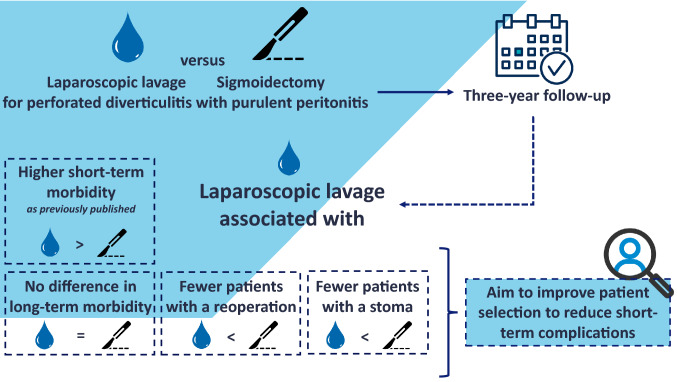

**Supplementary Information:**

The online version contains supplementary material available at 10.1007/s00464-022-09326-3.

Colonic diverticulosis is a common condition that affects up to 60% of people older than 60 years [1]. Approximately, 4–7% of colonic diverticulosis cases progress to diverticulitis [2, 3]. The prevalence of diverticulitis has increased over past decades and is estimated to be 180/100,000 persons per year [4]. In Western countries, diverticulitis has manifested itself as an expensive burden, being the fourth most costly diagnosed gastrointestinal disease in U.S. hospitals [5].

Up to 35% of patients with acute diverticulitis present with complicated disease, such as perforation with purulent or faecal peritonitis (Hinchey grade III or IV, respectively) [6]. Laparoscopic lavage has been introduced as alternative treatment for Hinchey grade III diverticulitis. Despite the recent publication of randomised controlled trials comparing sigmoidectomy and laparoscopic lavage, the role of lavage remains debated [7–11]. After laparoscopic lavage the sigmoid is left in situ with possible risk for short-term morbidity and recurrent diverticulitis [7, 12]. On the other hand, laparoscopic lavage might reduce the number of reoperations and stomas [13]. Long-term results of randomised controlled trials are of importance to determine whether these potential benefits outweigh the risk of short- and long-term complications.

Therefore, the aim of the present study was to assess long-term outcomes within the LOLA arm of the international, multicentre, randomised LADIES trial, in which laparoscopic lavage was compared to sigmoidectomy in patients with perforated diverticulitis with purulent peritonitis.

## Method

### Study design and participants

Long-term outcomes of patients included in the LOLA arm of the LADIES trial were assessed. The LADIES trial was a multicentre, parallel-group, open-label, randomised, superiority trial conducted in 34 teaching hospitals and eight academic hospitals in Belgium, Italy and the Netherlands. Patients between 18 and 85 years of age with signs of general peritonitis and suspected perforated diverticulitis were eligible for inclusion. Plain abdominal radiological examination or a CT scan had to show free intraperitoneal air- or fluid. Patients with dementia, previous pelvic irradiation, previous sigmoidectomy, treatment with high-dose steroids (> 20 mg daily) or preoperative shock requiring inotropic support were excluded, as well as patients with Hinchey I and II diverticulitis. Patients with purulent peritonitis without an overt perforation were randomly assigned (2:1:1) within the LOLA arm to receive laparoscopic lavage, Hartmann’s procedure or sigmoidectomy with primary anastomosis (with or without diverting ileostomy). This allowed a 1:1 comparison between laparoscopic lavage and sigmoidectomy. The study was designed in accordance with the Declaration of Helsinki and Good Clinical Practice guidelines. The ethical review board approved the study protocol in all participating hospitals. Before randomisation, written informed consent was obtained from all patients. The study protocol with further details on the study design, procedures, and outcome assessment was published previously, as well as the initial 12 month outcomes of the LOLA arm [7, 14]. The trial was registered with the Netherlands Trial Register (NTR2037) and ClinicalTrials.gov (NCT01317485).

### Long-term follow-up

In the present study, long-term outcomes were assessed up to 36 months after the index procedure. During the first 12 months, outcomes were collected prospectively. Additional follow-up data after the initial follow-up were retrospectively collected through review of patient’s medical records. All patients included in the LOLA arm were eligible for participation. Due to General Data Protection Regulation, patients who were still alive had to provide approval for the retrieval of long-term data and were contacted by either mail, or telephone, and by means of an information letter and study questionnaire. Patients who did not wish to participate or did not respond could not be included for long-term follow-up.

### Procedures

In general, to determine whether sigmoid perforation was present, any adherent tissue was carefully removed, but in case of firm adhesions they were left in place. In laparoscopic lavage, 6 L of warm saline were used to irrigate the abdominal cavity. Sigmoidectomy with primary anastomosis was performed according to the American Society of Colon and Rectal Surgeons guidelines and the decision to construct a diverting ileostomy was left to the surgeon’s preferences [15]. When allocated to Hartmann’s procedure, the diseased segment was dissected and the technique used to construct an end colostomy was chosen according to the preference of the operating surgeon. Further details of the surgical procedures including reinterventions and stoma reversals have been described previously [14].

### Outcomes

The primary outcomes of the present study were a composed endpoint of overall morbidity and mortality. Overall morbidity was defined as the occurrence of any of the following conditions or events: reinterventions (including surgical and percutaneous interventions, but excluding stoma reversal), abscess with drainage, abdominal wall complications (acute fascial dehiscence (ruptured abdomen) or parastomal/incisional hernia), recurrent diverticulitis, fistulae, and mortality. Recurrent diverticulitis episodes included uncomplicated and complicated cases. Complicated recurrent diverticulitis was defined as diverticulitis with the presence of a phlegmon, abscess, stenosis or perforation. Uncomplicated diverticulitis was registered if it was described in patient’s medical records, without the above described complications.

Secondary outcomes were the proportion of patients with one or more reoperations (surgical procedures related to the index procedure including stoma reversals), sigmoidectomy rates after initial treatment with lavage, stoma rates, percutaneous interventions, overall reinterventions, number of readmissions (including all readmissions without differentiation between related or not-related to index procedure)*,* total in-hospital days (index procedure, reversals, and readmissions combined), and sigmoid carcinomas. Overall reinterventions were defined as the combination of reoperation (including stoma reversal) and percutaneous interventions.

### Statistical analysis

Patients were analysed according to the intention-to-treat principle. Categorical data were presented as numbers with percentages. For comparison, the Fisher Exact test was applied. Continuous variables were presented as mean (standard deviation) or median (interquartile range) depending on distribution. If normally distributed, the *t*-test was applied to compare means. If not, the non-parametric Mann–Whitney *U*-test was used to compare medians. To analyse sigmoidectomy rates within the lavage group, sigmoidectomy-free survival was estimated with the Kaplan–Meier method. The percentage of patients with stoma and alive after 36 months was estimated with the Kaplan–Meier method, death within 36 months was censored. Difference in survival was analysed using the Mantel-Cox log-rank test.

## Results

### Study population

**Fig. 1:**
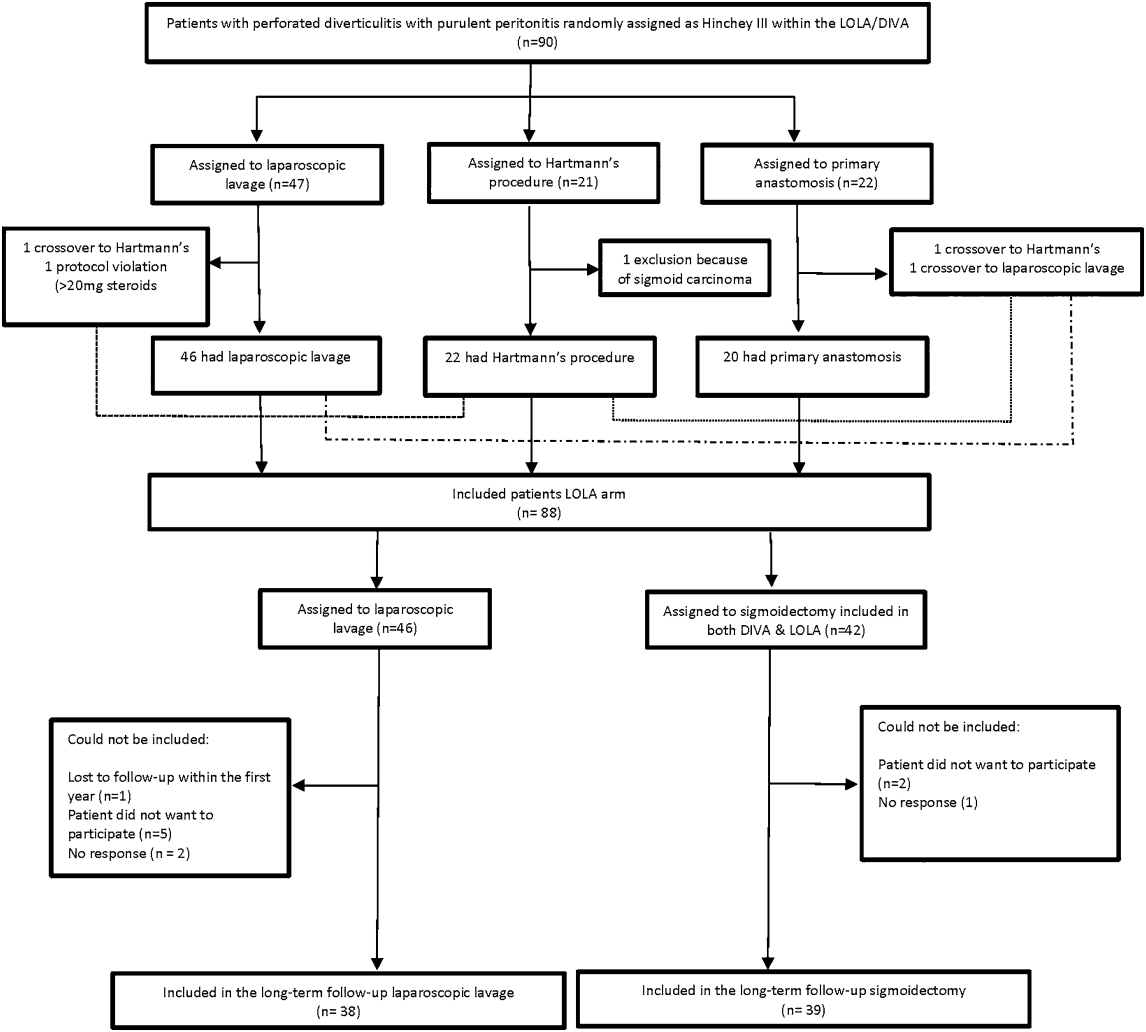
Trial profile long-term follow-up

A total of 88 patients were randomly assigned between July 1, 2010 and the early termination of the LADIES trial on Feb 22, 2013. Originally, 42 patients were assigned to sigmoidectomy and 46 to laparoscopic lavage. For the present 36 month follow-up, a total of 77 patients could be included, with 39 (51%) in sigmoidectomy group and 38 (49%) in the lavage group. Notably, 6 of 39 (15%) patients in the sigmoidectomy group and 4 of 38 (11%) in the lavage group died within the 12 month follow-up. After 12 months, 11 patients could not be included for the present long-term follow-up: refusal to participate (*n* = 7), no response (*n* = 3), lost to follow-up within the first 12 months (*n* = 1). The long-term trial profile is presented in Fig. 1.

### Baseline & (post)operative characteristics

As provided in Table 1, no major differences were observed between both sigmoidectomy and lavage in terms of baseline and (pre)operative characteristics.

**Table 1:** Baseline & (post)operative characteristics

	Sigmoidectomy (*n* = 39)	Laparoscopic lavage (*n* = 38)	*p*-value
Age (years)	63·9(12·3)	63·1(13·3)	0·955
Sex			0·494
Male	24(61·5)	20(52·6)	
Female	15(38·5)	18(47.4)	
Body-mass index (kg/m2) *	27·1(4·4)	27·5(6·6)	0·902
ASA			0.234
I	7(17·9)	8(21·1)	
II	12(30·8)	17(44·7)	
III	14(35·9)	5(13·2)	
IV	2(5·1)	3(7·9)	
Missing	4(10·3)	5(13·2)	
Previous diverticulitis*	9(25·7)	9(30·0)	0·784
Previous laparotomy*	3(7·7)	4(10·5)	0·854
Disease severity preoperative			
Mannheim peritonitis index	21(17–24)	21(17–25·3)	0·803
APACHE II score	8(6–12)	7(5–10·3)	0·163
POSSUM physiological score	21(18–27)	19·5(17–24·3)	0·224
POSSUM operative score	20(19–20)	17(17–17)	** < 0**·**001**
Interval from ER to surgery (h)*	12·5(6–42·3)	14(8–46)	0·631
(Post)operative characteristics			
Procedure			
Sigmoidectomy			
Primary anastomosis	20(48·7)	0	
Hartmann’s procedure	19(48·7)	1(2·6)	
Laparoscopic lavage	1(3·6)	37(97·4)	
Stoma constructed within the first year			
Ileostomy	12(30·8)	0	
Colostomy	20(51·3)	10(26·3)	
Stoma free after index procedure	7(17·9)	28(73·7)	
Operation time (min)	112(90–127)	58(46·75–95·75)	** < 0**·**001**
Number of patients operated on by a gastrointestinal surgeon	34(89·5)	33(86·8)	0·100

Significant outcome of *p* < 0.05 are given in bold

Data are mean (SD), n (%), or median (IQR)

*POSSUM* physiological and operative severity score for the enumeration of mortality and morbidity, *ASA* the American society of anesthesiologists, *APACHE* acute physiology and chronic health evaluation

*Occasional missing data

In the sigmoidectomy group, 20 of 39 patients (51%) underwent primary anastomosis procedure and 19 (49%) underwent a Hartmann’s procedure. One primary anastomosis patient crossed over to laparoscopic lavage due to the inability to fit in the stirrups after knee surgery. A colostomy was constructed in 20 of 39 patients in the sigmoidectomy group (51%) and a diverting ileostomy in 12 of 39 patients (31%). Seven of the 39 (18%) patients in the sigmoidectomy group had an anastomosis without diverting ileostomy. (Post) operative characteristics are presented in Table 1.

In this study, 4 of 38 (11%) patients in the laparoscopic lavage group and 2 of 39 (5%) patients in the sigmoidectomy group were diagnosed with a sigmoid carcinoma (*p* = 0·431).

### Primary outcome

Table 2 presents the main outcomes of the present study. The composite endpoint showed no differences in cumulative morbidity (sigmoidectomy 28/39 (72%) versus lavage 32/38 (84%), *p* = 0·272), further specification of morbidity is presented in Table 3. In terms of 36 month mortality, no difference was observed between sigmoidectomy and lavage (7/39 (18%) versus 6/38 (16%), *p* = 1·000), cause of death is specified in Supplementary Table 1. In addition, morbidity during the 12–36 month follow-up period was not significantly different between both groups either (sigmoidectomy 7/33 (22%) versus lavage 9/34 (27%), *p* = 0·776) (Supplementary Table 2).

**Table 2:** Main outcomes 36 months after index procedure

	Sigmoidectomy (*n* = 39)	Laparoscopic lavage (*n* = 38)	*p*-value
Overall morbidity	28(71·8)	32(84·2)	0·272
Mortality	7(17·9)	6(15·8)	1·000
Patients with ≥ 1 reoperation, n(%)	27(69·2)	17(44·7)	0·039
Patient alive with stoma in situ*	4(17·0)	4(10·5)	0.0268^†^
Stomas not reversed	11(28·2)	4(10·5)	0·083
Sigmoid resection after allocation to laparoscopic lavage		17(44·7)	
Reoperations per patient, n (%)			0·082
0	12(30·8)	21(55·3)	
1	18(46·2)	8(21·1)	
2	5(12·8)	6(15·8)	
≥ 3	4(10·3)	3(7·9)	

Data are n (%) or median (IQ), *p*-value is the outcome of the fisher exact-test *Cumulative proportion calculated from the Kaplan–Meier life table

^†^*p*-value from log-rank test

**Table 3:** Overall morbidity and mortality 0–36 months after index procedure

	Sigmoidectomy (*n* = 39)		Laparoscopic lavage (*n* = 38)		*p*-value
	Patients	Events	Patients	Events	
Overall morbidity	28(71·8)	27	32(84·2)	39	0·272
Reintervention	13(33·3)	14	21(55·3)	31	
Surgical	12(30·8)	13	17(44·7)	30	
Percutaneous	1(2·6)	1	1(2·6)	1	
Abscess with drainage	1(2·6)	1	8(21·1)	15	
Abdominal wall complications	10(25·6)	11	5(13·2)	6	
Incisional/parastomal hernia	7	8	4	4	
Fascial dehiscence	3	3	0	0	
Recurrence diverticulitis	1(3·4)	1	8(21·1)	8	
Fistula	0(0)	0	2(5·3)	2	
Mortality	7(17·9)	7	6(15·8)	6	1·000
Related to surgery or diverticulitis	1		2		
Unrelated	5		3		
Cause unknown	1		1		

Data are n(%), *p*-values are from numbers of patients, not event numbers. Cause of death specified in Supplementary Table 4

### Reoperation

After 36 months, the number of patients who underwent a reoperation was significantly lower in the laparoscopic lavage group compared to the sigmoidectomy group (sigmoidectomy 27/39 (69%) versus lavage 17/38 (45%), *p* = 0·039) (Table 2).

In the sigmoidectomy group, 27 patients were reoperated reoperations with a total of 36 events; stoma reversal (*n* = 23), abdominal wall complication requiring surgical repair (*n* = 6), post-operative complications requiring surgical intervention (*n* = 6), and recurrent diverticulitis that led to surgical intervention (*n* = 1). 9 of 36 (25%) reoperations were performed in an emergency setting.

In the lavage group, 17 patients were reoperated with a total of 35 events as well; sigmoidectomy (*n* = 17), stoma reversal (*n* = 6), post-operative complications requiring surgical intervention (*n* = 9), abdominal wall complication requiring surgical repair (*n* = 2), and reoperation due to metastasised sigmoid carcinoma (*n* = 1). 15 out of 35 (43%) reoperations were performed in an emergency setting of which two patients were responsible for 8 emergency operations all performed shortly after index procedure. Further details of the reoperations are listed in Supplementary Table 3.

### Sigmoidectomy and recurrent diverticulitis rates after lavage

**Fig. 2:**
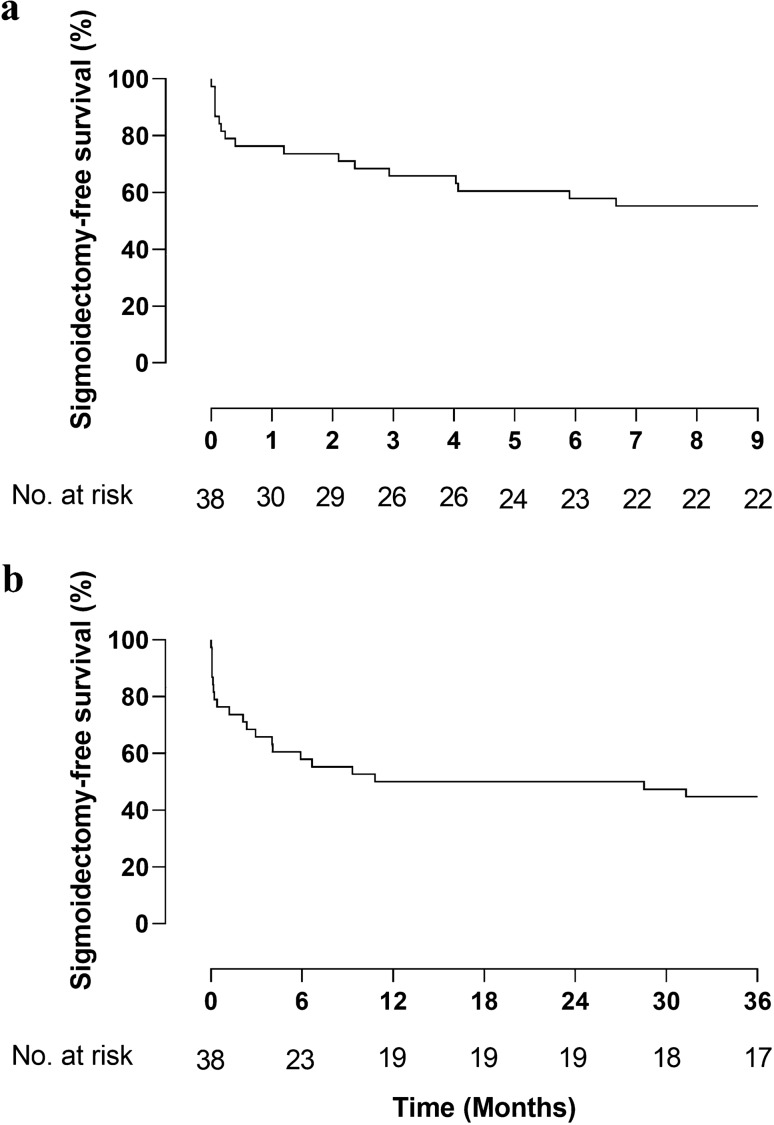
**a** and **b** Kaplan–Meier graph of 9 month (**a**) and 36 month (**b**) sigmoidectomy-free survival in patients allocated to laparoscopic lavage

A total of 17 patients (45%) did not undergo sigmoidectomy and were alive after 36 months (Fig. 2). Four patients died with their sigmoid in situ. Eventually, 15 of 17 sigmoidectomies (88%) were performed within the first 12 months.

Four patients underwent sigmoidectomy for sigmoid carcinoma (Hartmann’s procedure (*n* = 3) and sigmoidectomy with primary anastomosis (*n* = 1)). Two patients were diagnosed during follow-up colonoscopy. In one patient, the malignancy was the cause of immediate failure of lavage to control sepsis. The other patient was diagnosed at eight months after presentation with a colovesical fistula.

Failure to control sepsis required emergency reoperation and sigmoidectomy in six patients (all six patients underwent Hartmann’s procedure).

Two patients had recurrent abdominal complaints that gave reason to perform elective sigmoidectomy with primary anastomosis (within one year after the index procedure).

Eventually, eight of 38 (21%) patients treated with lavage were diagnosed with recurrent diverticulitis. Four patients underwent sigmoidectomy with primary anastomosis (two within the first year, one after 2 years and one after 3 years). Four patients having recurrent diverticulitis were treated conservatively. Further details of the sigmoidectomies are listed in Supplementary Table 3.

### Stoma rates

**Fig. 3:**
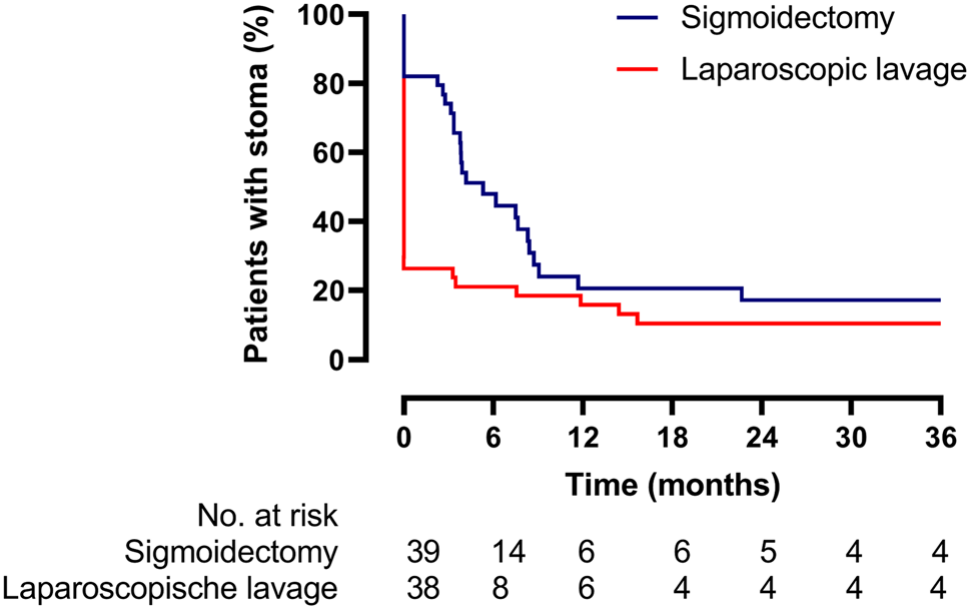
Kaplan–Meier graph of patients alive and with stoma in situ after 36 months

After 36 months, the percentage of patients alive with stoma in situ was significantly lower for those who underwent lavage compared with sigmoidectomy (cumulative proportion calculated from the Kaplan–Meier life table, sigmoidectomy 17% vs lavage 11%, log-rank *p* = 0·0268) (Fig. 3). This only included colostomas and no ileostomas were in situ at this time point. In ten of 38 patients (26%) in the laparoscopic lavage group a colostomy was constructed for the following reasons: failure to control sepsis (*n* = 6), sigmoidectomy with colostomy for sigmoid carcinoma (*n* = 3), Hinchey 4 diagnosis during surgery after being allocated to lavage (*n* = 1).

In the sigmoidectomy group, seven of 39 patients (18%) underwent sigmoidectomy with primary anastomosis and no stoma was constructed.

In the lavage group, 28 of 38 patients (73%) never had a stoma after 36 months of whom seven of 28 patients (25%) underwent sigmoidectomy with primary anastomosis.

Reasons not to reverse are listed in Supplementary Table 4.

### Secondary outcomes

The proportion of patients requiring percutaneous drainage was higher in the lavage group (sigmoidectomy 2/39 (5%) versus lavage 9/38 (24%), *p* = 0·025). Consequently, the overall reintervention rate did not statistically differ (sigmoidectomy 27/39 (69%) versus lavage 21/38 (55%), *p* = 0·798). No difference in median number of procedures, readmissions and total hospital days was observed (Table 4).

**Table 4:** Secondary outcomes within 36 months

	Sigmoidectomy (*n* = 39)	Laparoscopic lavage (*n* = 38)	*p*-value*
Median reoperations per patient (IQ)	1(0–1)	0(0–1·25)	0·143
Patients with ≥ 1 percutaneous intervention, *n*(%)	2(5·1)	9(23·7)	**0**·**025**
Patients with ≥ 1 Overall reintervention, *n*(%)	27(69·2)	21(55·3)	0·244
Readmission per patient			0·392
0	7(17·9)	12(31·6)	
1	14(35·9)	8(21·1)	
2	11(28·2)	10(26·3)	
≥ 3	7(17·9)	8(21·1)	
Median of readmissions per patient (IQ)	1(1–2)	1(0–2)	0·733
Total duration of hospital stay per patient (days)	19(15–31)	18(8·75–36·75)	0·429

Significant outcome of *p* < 0.05 is given in bold

Data are n (%) or median (IQ)

*p-value is the outcome of the fisher exact-test

## Discussion

In the present long-term follow-up comparing laparoscopic lavage with sigmoidectomy in patients with perforated diverticulitis with purulent peritonitis (LOLA arm of the randomised LADIES trial), it was found that after 36 months overall morbidity and mortality did not differ between both procedures.

However, the number of patients who underwent a reoperation was lower if the patient had been treated with laparoscopic lavage. Additionally, the percentage of patients alive with stoma in situ was lower for those who underwent lavage compared with sigmoidectomy.

In line with these results, the DILALA trial did also show the benefit of lavage in terms of reoperations during a 24 month follow-up period (lavage 18/43 versus resection 27/40, *p* = 0.01) [13]. In addition, the long-term results of the SCANDIV trial did not show a significant difference in the number of reoperations (lavage 26/73 versus resection 24/69, p = 0·92) [11]. In terms of overall morbidity, no differences between both groups were observed in the SCANDIV long-term outcomes and the present study [11]. Importantly, in patients alive after 36 months fewer stoma rates were found in the lavage group compared to the sigmoidectomy group, which was confirmed by the long-term DILALA and SCANDIV results [11, 13]. Although the difference was significant in the present study, this was mainly determent by the fact that in the lavage group the period of time with a stoma was lower as the majority never had a stoma. Eventually, stomas in the sigmoidectomy group were reversed which resulted in comparable rates after 36 months.

As described previously, leaving the affected sigmoid in situ resulted in more short-term complications in the LOLA 12 months follow-up. Although the SCANDIV- and DILALA trial did not find excessive short-term morbidity [7, 16]. Failure to control sepsis in a patient with perforated diverticulitis might cause these short-term complications, especially since reoperations or percutaneous drainage could be indicated. The superior long-term outcomes of laparoscopic lavage need to be weighed against the potential increased short-term risks. Hence, reduction of short-term morbidity becomes of even greater importance as this might shift this balance. Preoperative characteristics (such as age, ASA grade, comorbidities, and inflammation parameters) might help to select patients that are prone to failure of lavage (e.g. short-term sigmoidectomy). In the present study, no discriminative analyses could be performed due to a lack of power. Moreover, improvement of pre- or perioperative diagnostics is of importance to better discriminate between Hinchey III diverticulitis and Hinchey IV or underlying carcinomas also. To our opinion, this could for example be achieved through the introduction of a CT-scan with rectal contrast in all patients suspected for complicated diverticulitis. Perioperative solutions might include a sigmoidoscopy, hydro-pneumatic testing or introduction of rectal contrast (e.g. methylene blue) to differentiate diverticulitis from perforated cancer and a Hinchey III from IV [17–21].

The present study also focused on reoperations after index procedure with the inclusion of stoma reversals as this directly highlights the difference with sigmoidectomy with stoma construction, subsequent stoma reversal, and potential reversal- or stoma-related morbidity (e.g. parastomal/incisional hernia). Moreover, stoma reversal should not be underestimated in terms of associated risk of morbidity and mortality [22]. The authors recognise that reoperations in lavage group are unplanned compared to planned reversals in the sigmoidectomy group. However, patients who underwent unplanned elective operations are not by definition more at risk than those undergoing colo/ileostomy reversals. Therefore, the number of emergency operations were considered which was higher in the lavage group. In the lavage group, the majority of these events occurred shortly after index procedure which confirms the short-term risk and the importance of patient selection.

Six of 77 (8%) patients were diagnosed with sigmoid carcinoma. The prevalence of colorectal carcinomas seems to be higher in patients suspected for complicated diverticular disease compared to uncomplicated disease [23, 24]. Therefore, a colonoscopy (e.g. after at least 6 weeks) after the initial treatment is recommended for all patients with complicated diverticulitis and is even of more importance when treated with lavage [23–25].

It has to be considered that leaving the sigmoid in situ could result in recurrent diverticulitis. In our study, 21% of patients treated with laparoscopic lavage had a recurrent episode within 36 months which included episodes of Hinchey I or II only. Eventually, 50% underwent elective sigmoidectomy with primary anastomosis. Long-term outcomes of the SCANDIV trial found a recurrence rate of 21% [11]. Retrospective cohort studies following patients treated for perforated diverticulitis with laparoscopic lavage showed recurrence rates up to 30% [12, 26].

Patients who suffered from ongoing abdominal complaints or recurrent diverticulitis on the long-term underwent an elective sigmoidectomy with primary anastomosis in most cases. This indicates that in selected patients, lavage could also be useful to overcome an emergent operation. If sigmoidectomy is still indicated on the long-term, an elective laparoscopic resection with primary anastomosis is the preferred treatment which preserve patients of stoma construction.

There were limitations to this study. Within the LADIES trial, a LOLA- and DIVA arm was included. Therefore, the sigmoidectomy group consisted of Hartmann’s procedure and sigmoidectomy with primary anastomosis (with or without ileostomy), which could have affected the stoma reversal rate and reoperation rate [27]. Morbidity and mortality rates were not expected to be affected since no differences were found in previous randomised controlled trials [27–29].

In addition, attrition bias might have been introduced due to General Data Protection Regulations requiring patients to provide approval for retrieval of data from patients records. However, loss to follow-up was relatively low and equally distributed among randomised groups. Therefore, any effects of loss to follow-up are likely not differential.

In conclusion, long-term outcomes showed that laparoscopic lavage was associated with less patients who underwent a reoperation and lower stoma rates in patients alive after 36 months compared to sigmoidectomy. No differences were found in terms of cumulative morbidity or mortality. These long-term benefits need to be weighed against the evidential risk of short-term complications, especially on going sepsis should be avoided as this could be a potentially fatal condition. These short-term complications could be further reduced by the optimisation of patient selection, pre-, and perioperative diagnostics. To define the optimal treatment, patients should be involved in the decision-making process after being informed about short- and long-term consequences regarding lavage. In that context, lavage could be valuable as treatment for perforated purulent diverticulitis.

## Supplementary Information

Below is the link to the electronic supplementary material.

Supplementary file1 (DOCX 24 kb)
