# Supplementary material for: Laparoscopic peritoneal lavage versus sigmoidectomy for perforated diverticulitis with purulent peritonitis: three-year follow-up of the randomised LOLA trial
Source: Surg Endosc. 2022 May 23;36(10):7764–74. doi: 10.1007/s00464-022-09326-3 (PMC9485102; doi:10.1007/s00464-022-09326-3)
Supplement: Supplementary file 1 — Supplementary file1 (DOCX 24 kb) [file 464_2022_9326_MOESM1_ESM.docx]

| **Supplementary Table 1; Cause of death** |  |
| --- | --- |
| **Laparoscopic lavage (n=6)** | **Patients (n=)** |
| Unknown | 1 |
| Fatal head trauma (unrelated) | 1 |
| Myocardia infarct >30 days after surgery (unrelated) | 1 |
| Myocardia infarct <30 days after surgery (related) | 2 |
| Lung carcinoma (unrelated) | 1 |
| **Sigmoidectomy (n=7)** | **Patients (n=)** |
| Unknown | 1 |
| Pneumosepsis after femur amputation (unrelated) | 1 |
| Lung carcinoma (unrelated) | 1 |
| Arterial occlusion after index procedure (related) | 1 |
| Myocardia infarct >30 days after surgery (unrelated) | 1 |
| Kidney failure >30 days after surgery (unrelated) | 1 |
| Metastasized prostate cancer (unrelated) | 1 |

| **Supplementary Table 2; Morbidity outcomes 0-36 months after index procedure** | | | |  |  |
| --- | --- | --- | --- | --- | --- |
|  | **Sigmoidectomy  (n=39)** | | **Laparoscopic lavage  (n=38)** | | P-value |
|  | Patients | Events | Patients | Events |  |
| **0-12 months morbidity** |  |  |  |  |  |
| Overall morbidity | 17(43·6) |  | 24(63·2) |  | 0·111 |
| Reintervention* | 8(20·5) | 9 | 19(50) | 27 |  |
| *Surgical* | 7(17·9) | 7 | 16(42·1) | 27 |  |
| *Percutaneous* | 2(5·1) | 2 | 1(2·6) | 1 |  |
| Abscess with drainage | 0(0) | 0 | 7(18·4) | 14 |  |
| Abdominal wall complications | 6(15·4) | 6 | 3(7·9) | 4 |  |
| *Incisional/parastomal hernia* | 3 | 3 | 2 | 2 |  |
| *Fascial dehiscence* | 3 | 3 | 0 | 0 |  |
| Recurrence diverticulitis | 0(0) | 0 | 4(10·5) | 4 |  |
| Fistula | 0(0) | 0 | 1(3·6) | 1 |  |
| Mortality | 6(15·4) | 6 | 4(10·5) | 4 |  |
|  |  |  |  |  |  |
| **12-36 months morbidity** | Sigmoidectomy (n=33) |  | Laparoscopic lavage (n=34) |  |  |
| Overall morbidity | 7(21·2) |  | 9(26·5) |  | 0·776 |
| Reintervention | 5(15·2) | 6 | 3(8·8) | 3 |  |
| *Surgical* | 5(15·2) | 6 | 3(8·8) | 3 |  |
| *Percutaneous* | 0(0) | 0 | 0(0) | 0 |  |
| Abscess with drainage | 0(0) | 0 | 1(2·9) | 1 |  |
| Abdominal wall complications | 5(15·2) | 5 | 2(5·9) | 2 |  |
| *Incisional/parastomal hernia* | 5 | 5 | 2 | 2 |  |
| *Fascial dehiscence* | 0 | 0 | 0 | 0 |  |
| Recurrence diverticulitis | 1(3·0) | 1 | 4(11·8) | 4 |  |
| Fistula | 0(0) | 0 | 1(2·9) | 1 |  |
| Mortality | 1(3·0) | 1 | 2(5·9) | 2 |  |
|  |  |  |  |  |  |
| **Overall morbidity** | 28(71·8) | 27 | 32(84·2) | 39 | 0·272 |

Data are n (%), P-values are from numbers of patients, not event numbers. Overall morbidity includes both the 0-12 months plus 12-36 months follow-up.

| **Supplementary Table 3; Reoperations specified** |  |  |  |  |
| --- | --- | --- | --- | --- |
| **Laparoscopic lavage (n=17)** | **Events** | Elective | Emergency |  |
| **Sigmoidectomy*** | **18** |  |  |  |
| *Recurrent abdominal complaints but not diagnosed as diverticulitis* | *2* | *2* | *0* |  |
| *Recurrent diverticulitis* | *4* | *3* | *1* |  |
| *Failure of lavage and persisting sepsis*‡ | *5* | *0* | *5* |  |
| *Failure of lavage caused by missed/diagnosed Hinchey 4 diverticulitis* | *2* | *0* | *2* |  |
| *Hinchey 4 diagnosis during index procedure after allocation* | *1* | *-* | *-* |  |
| *Treatment for sigmoid carcinoma* | *4* | *3* | *1* |  |
| **Post-operative complications requiring surgical intervention** | **9** |  |  |  |
| *Additional lavage after failure to control sepsis (four times repeated in one patient)* | *5* | *0* | *5* |  |
| *One patient underwent Hartmann’s procedure without resection, lavage and stump resection prior to sigmoidectomy* | *3* | *1* | *2* |  |
| *HIPEC* | *1* | *1* | *0* |  |
| **Stoma reversal†** | **6** | 6 | 0 |  |
| **Abdominal wall complication with surgical repair** | **2** |  |  |  |
| *Fascial dehiscence* | *0* | *0* | *0* |  |
| *incisional/parastomal hernia* | *2* | *2* | *0* |  |
| **Metastasis requiring reoperation** | **1** | 1 | 0 |  |
| **Sigmoidectomy (n=27)** | **Events** | Elective | Emergency |  |
| **Stoma reversal+** | **23** | 23 | 0 |  |
| **Abdominall wall complication with surgical repair** | **6** |  |  |  |
| *Fascial dehiscence* | *3* | *0* | *3* |  |
| *incisional/parastomal hernia* | *3* | *3* | *0* |  |
| **Post-operative complications requiring surgical intervention** | **6** |  |  |  |
| *Intra-abdominal bleeding with unknown origin* | *1* | *0* | *1* |  |
| *Anastomotic leakage* | *1* | *0* | *1* |  |
| *Stenotic colonic anastomosis without passage* | *1* | *0* | *1* |  |
| *Abscesses near spleen after Hartmann’s procedure requiring splenectomy* | *1* | *0* | *1* |  |
| *Lavage due to intraabdominal abscesses after stoma reversal* | *1* | *0* | *1* |  |
| *Relaparatomy by infected hematoma/abscess after stoma reversal* | *1* | *0* | *1* |  |
| **Left-hemicolectomy due to recurrent diverticulitis** | **1** | 1 | 0 |  |

* in one patient a sigmoid carcinoma led to failure of lavage and was scored in both groups (failure of lavage and treatment for sigmoid carcinoma); † in one patient a stoma reversal was complicated by anastomotic leakage requiring reintervention and colostomy construction, the colostoma was reversed 1.5 year later and two events were added to stoma reversal;

| **Supplementary Table 4; Reasons stomas were not reversed.** |  |
| --- | --- |
| **Laparoscopic lavage (n=4)** | **Patients (n=)** |
| Due to discovery of an adenocarcinoma in the sigmoid. | 1 |
| Oncological resection and HIPEC (and the patient was not seen for reversal afterward). | 1 |
| loco-regional metastases. | 1 |
| Patient chose not to reverse. | 1 |
| **Sigmoidectomy (n=11)** | **Patients (n=)** |
| Died having a stoma in situ. | 7 |
| Due to vascular comorbidities and diabetes. | 1 |
| Stoma reversal was extended due to prioritised incisional hernia repair. | 1 |
| Left-hemicolectomy with colostomy was performed after recurrent diverticulitis was diagnosed. | 1 |
| Patient chose not to reverse. | 1 |
